# Effectiveness of Enhanced Self-Positivity Bias Training in Mitigating Depressive Mood following Negative Life Events

**DOI:** 10.3390/bs13070534

**Published:** 2023-06-27

**Authors:** Lingyun Wang, Xiaoling Song, Fanli Jia, Wenxia Xue, Lihong Li

**Affiliations:** 1 School of Psychology, Northeast Normal University, Changchun 130024, China; 2 Jilin Provincial Key Research Base of Humanities and Social Sciences (Northeast Normal University) Mental Health Education Research Center, Changchun 130024, China; 3 College Students’ Mental Health Education and Consultation Center, Lanzhou Resources Environment Voc Tech University, Lanzhou 730021, China; 4 Department of Psychology, Seton Hall University, South Orange, NJ 07079, USA; 5 The Second Experimental School of Jilin Province, Changchun 130012, China; 6 Changchun Humanities and Sciences College, Northeast Normal University, Changchun 130119, China

**Keywords:** self-positivity bias, depressive mood, self-reference task, negative life events

## Abstract

The phenomenon of self-positivity bias refers to the common tendency for individuals to perceive themselves in a more positive light than is objectively warranted. The current study seeks to investigate the impact of enhanced self-positivity bias on depressive mood resulting from negative life events. The study included two experiments, a resistance experiment (exp. 1) and an improvement experiment (exp. 2), with 40 randomly selected college students randomly assigned to either a self-positive bias training group or a neutral training group in each experiment. In the resistance experiment, self-positive bias training was conducted before failure feedback, while in the improvement experiment, it was conducted after failure feedback. The results showed that failure feedback significantly increased depression levels among college students, and self-positive bias training improved the level of self-positive bias. In the resistance experiment, there was no significant difference between the self-positive bias training group and the neutral training group regarding depression. However, in the improvement experiment, being in the self-positive bias training group had a significantly greater effect on improving depression compared to the neutral training group. Overall, the findings suggest that while self-positive bias training cannot prevent depression caused by failure events, it has a positive effect on improving depression.

## 1. Introduction

Individuals may encounter various negative events throughout their lives that elicit negative emotional distress, such as temporary depressive mood [1,2]. When the negative emotional distress is not able to be coped with over time, it may develop into a long-lasting depression [3]. In addition, normal individuals exhibit a self-positivity bias, in which they attribute positive aspects to their own stable personality traits, resulting in the perception of greater competence, kindness, and superiority [4,5,6]. According to self-affirmation theory, individuals have a fundamental desire and motivation to maintain their self-integrity [5,6]. The ability to perceive oneself as competent, conforming to societal norms, and in control of one’s life can help individuals to promote positive psychological wellbeing, even when they encounter self-esteem threats such as failure [7]. By activating or enhancing positive self-bias, it is possible to help individuals cope with emotional distress and improve their psychological well-being.

The concept of self-positivity bias consists of both implicit and explicit components. Implicit self-positivity bias involves an automated positive evaluation of oneself that occurs without conscious awareness, and its intrinsic mechanisms may be related to implicit self-esteem [8,9]. The Implicit Association Test (IAT) is the most dominant paradigm for measuring implicit self-esteem because it measures the degree of association between the self and positive adjectives. While explicit self-esteem is strongly predictive of psychological well-being, physical and mental health, temporary mood, and depression, implicit self-esteem does not have such a strong effect [10]. As a result, implicit and explicit self-esteem indicates different aspects of the self. In an experiment similar to the implicit self-esteem test, subjects were asked to rate the adjectives of personality traits presented following the words “I” or “he” in a positive or negative manner, and when subjects responded more quickly to the “I” and positive adjective pairs than other pairs, implicit self-positivity bias was present. The implicit bias in favor of self-positivity is prevalent among normal individuals but disappears when a person is depressed [11,12,13].

Moreover, researchers have examined various manipulations intended to enhance implicit self-esteem/implicit self-positivity bias in an effort to understand their mechanisms. For example, subliminal evaluative conditioning tasks in which positive trait words were repeatedly presented with the word “I” promoted implicit self-positivity bias [11] or implicit self-esteem [14,15]. When participants’ implicit self-esteem is enhanced, they are less sensitive to negative intelligence feedback, which leads to them feeling less depressed after a general failure event [15]. Nevertheless, one study asked participants in a group to perform a self-reference task that classified four types of stimuli (me versus others, pleasant versus neutral) into two categories: me or pleasant, others or neutral. Compared with the control group, the experimental group had higher explicit self-esteem and a higher explicit positive self-view, but there was no difference in implicit self-esteem. This manipulation, however, did not affect individuals’ psychological well-being (measured using the Anxiety, Depression, and Stress Scale) [16]. Training programs based on conditioned learning or self-reference tasks do not always increase implicit self-esteem or implicit positive self-bias or consistently improve social outcome variables such as depression. As a result, it may be more appropriate to develop training programs that enhance explicit self-positivity bias in order to reduce stress and depression caused by negative events.

Explicit self-positivity bias is typically evident at the conscious level. During conscious self-appraisal processes, individuals tend to hold a positive view of their own personality. For example, when asked to assess the extent to which positive and negative personality trait adjectives describe themselves and others, the majority of individuals perceive positive traits as more applicable to themselves, while disregarding negative traits [6]. Clearly, to enhance the explicit self-positivity bias, the manipulations previously applied to the enhancement of implicit self-positivity bias may not be applicable, as conditioned learning is implicit and self-esteem is irrelevant at both implicit and explicit levels. In other words, improvement of implicit self-esteem may not necessarily lead to an improvement in explicit self-esteem. McAdams et al. showed that traits and life narratives are two different dimensions of personality and that the characteristics of narrative stories provide evidence for changes in personality [17]. While simultaneously transforming the meaning of life narrative events as autobiographical memory is associated with self-transformation, positive narrative processes can facilitate positive self-development [17,18]. By reflecting on positive experiences, individuals can enhance their identification with the positive self. In particular, this approach can promote the development of a positive self-image and reduce negative emotional distress.

In light of the above review, it appears that the key aspect of the procedure to increase positive self-bias is to link positive self traits with the conscious extraction of autobiographical memories. Following the association, the self-positivity bias would be strengthened against depressive mood caused by stress. Furthermore, a modified self-reference task similar to the IAT has been shown to be effective in enhancing individuals’ explicit positive self-view [16]. We further modified this task by training participants in self-positivity bias, in which the self and positive trait adjectives were paired, and participants were asked to judge to what extent they were similar to the positive trait adjectives. In this modified self-reference task, positive words were presented only to increase explicit self-positivity bias, creating a positive environment for the participants and prompting self-reflection by asking participants to examine how similar they were to the positive words [19]. We anticipated that this task may enhance positive self-bias by assisting the individual in continuously associating positive traits with oneself.

The present study investigated whether explicit self-positivity bias training enhanced self-positivity bias and whether enhanced self-positivity bias could moderate depressive mood triggered by a negative event. In this study, two experiments were used to illustrate the effects of enhanced self-positive bias on depressive mood in two differing ways. During experiment 1, participants were trained to enhance self-positivity bias before receiving negative feedback. The aim of this study was to investigate whether enhancing self-positivity bias could help counteract depressive mood caused by failure. In Experiment 2, participants were trained on self-positivity bias following failure in order to determine if enhancing self-positivity bias could alleviate failure-induced depression. It was hypothesized in this study that self-positivity bias training would aid in increasing people’s self-positivity bias as well as preventing and improving depressive moods.

## 2. Experiment 1: Resistance to Depressive Mood via Self-Positivity Bias Training

### 2.1. Method

#### 2.1.1. Participants

Forty college students, 9 men and 31 women, aged 18–25 years (mean age 21 ± 2.3) were randomly selected at Northeast Normal University. We randomly assigned 20 participants to each of the experimental and control groups. Prior to participating in the experiment, all participants had normal or corrected visual acuity, had not previously taken the Raven’s Standard Progressive Matrices test in other studies, and had not experienced failure-induced feedback. They provided informed consent before beginning the experiments and received appropriate compensation upon completion.

#### 2.1.2. Experimental Apparatus

The participants in the study underwent testing and training using a laptop computer equipped with a 15″ monitor and a screen resolution of 1024 × 768. The failure feedback was generated and presented using the website www.eqxiu.com accessed on 1 September 2019. The measurements of self-positivity bias, self-positivity bias training, and neutral training programs were designed and implemented using E-prime 2.0.

#### 2.1.3. Measurements

The Depressive Mood Scale was based on the Depression Adjective Check List (DACL) developed by Lubin [20]. The F scale was used to assess temporary or transient depression. There are 34 mood words on this scale, 22 of which referred to a depressed state and 12 to a non-depressed state. The participants were asked to indicate whether the mood word described their current state of mind. In both cases, a score of one was given for either the depressed word or the non-depressed word, with higher scores indicating greater severity of depression [21]. Additionally, the 34 mood words were randomly arranged into three sequences for the depressive mood pre-test, mid-test, and post-test to reduce the effects of subject memory.

Self-positivity bias. A total of 80 adjectives were selected from 562 personality trait adjectives compiled by Huang et al. [22], with 40 being positive and 40 negative. These words were rated by five psychology postgraduates on their goodness, familiarity, and meaning. The results showed that there were significant differences between the two groups of words in terms of their goodness and badness, but not in terms of familiarity and meaning. During the experiment, the 80 selected adjectives were presented on a computer screen, and the participants were asked to judge the extent to which these words described themselves (the extent to which they were like or unlike themselves). Each response was scored on a 6-point Likert scale, from “very unlike” to “very much like”. The total scores for positive words and negative words that described themselves were calculated separately. If the total score for positive words was significantly higher than the total score for negative words, it indicated that the individual had a self-positivity bias. The difference between the positive and negative scores was used as the score of self-positivity bias.

False failure feedback. We selected the 10 most difficult questions from the Raven’s Standard Progressive Matrices (questions A12, B12, C12, D12, E12, A11, B11, C11, D11, E11). Participants were given seven minutes to answer the questions carefully. The results of the test were presented on a website (www.eqxiu.com accessed on 1 September 2019) to enhance the credibility of the results. Prior to the test, we informed the participants that they would be required to take an official intelligence test consisting of 10 questions within seven minutes. Upon submission, the website background calculates the score and provides the obtained score and answer analysis. After completing the test, each participant received the same feedback. The message read, “____ marks. Explanation: You have failed this test and your correct score is below the average for university students (on average, a Chinese university student gets seven questions correct)”. This method has been used in previous studies to effectively induce feelings of failure [23].

Self-positivity bias and neutral training procedures. We selected other 80 positive words and 80 neutral words from 562 personality trait adjectives compiled by Huang et al. [22]. We used these words in the self-positivity training group and in the neutral training group. These words were not the same as the ones used to measure self-positivity bias. During the training process, a “+” appeared in the center of the screen for 250 ms, followed by the phrase “I + adjective” and four options (1: not at all like; 2: somewhat like; 3: more like; 4: very much like). The participants were asked to choose the option that was closest to them. The word disappeared after the subject made their choice, and the participants moved on to the next trial. Each word was presented once, for a total of 80 training sessions.

#### 2.1.4. Experimental Procedures

The experiment consisted of three testing phases and three manipulation phases. The first phase was the pre-test, where the participants’ depressive mood and self-positivity bias were measured. Then, the participants underwent self-positivity bias training or neutral training during the second phase. After the training, the mid-test was conducted to measure the participants’ depressive mood and self-positivity bias again. Next, they received false failure feedback during the third phase. The post-test was then conducted to measure the participants’ depressive mood. Finally, the participants had a debriefing discussion with the researcher to eliminate any negative effects of the false feedback on them. The participants were informed that the Raven reasoning test was too difficult, and the failure feedback was inaccurate as the study was not intended to measure reasoning ability.

### 2.2. Results

#### 2.2.1. Testing the Validity of Self-Positivity Bias

Table 1 shows the means and standard deviations of self-positivity bias scores in pre-test and post-test. To test the effectiveness of self-positivity bias training, a repeated measures analysis of variance with 2 groups (experimental, control) × 2 measurement times (pre-test, post-test) was conducted on the self-positivity bias scores. The results found that the interaction between group and measurement time was significant, *F*(1,38) = 9.17, *p* < 0.01, *η_p_*^2^ = 0.194. Simple effects analysis revealed that the self-positivity bias scores of the experimental group were significantly higher on the post-test than on the pre-test (*t* = 3.016, *p* < 0.01, d′ = 0.674). However, the difference between the self-positivity bias scores of the control group on the pre-test and post-test was not significant (*p* = 0.635), suggesting that self-positivity bias training was effective in enhancing self-positivity bias, whereas neutral training did not change self-positivity bias.

#### 2.2.2. Analysis of the Effect of Self-Positivity Bias Training on Resistance to Post-Failure Depressive Mood

Table 2 gives the means and standard deviations of depressive mood scores between groups and times in Experiment 1. An analysis of variance with repeated measures was conducted on the depressive mood scores for 2 groups (experimental, control) and 3 measurement times (pre-test, mid-test, post-test). A significant main effect of measurement time, *F*(2,76) = 25.60, *p* < 0.001, *η_p_*^2^ = 0.402, was found. Post-hoc analysis showed that depressive mood scores were significantly higher in the post-test than in the pre-test and mid-test (*ps* < 0.001), and there were no significant differences between the pre-test and mid-test (*p* = 0.768). The results showed that the false failure feedback did induce depressive mood in the participants, suggesting that the manipulation was effective. However, the main effect of the group was not significant *F*(1,38) = 1.30, *p* = 0.261, indicating that there was no significant difference in the level of depressive mood between the experimental and control groups overall. Additionally, the interaction effect between the measure and group was not significant *F*(2,76) = 0.20, *p* = 0.819, indicating that self-positivity bias training did not have a significant impact on reducing the level of depressive mood caused by the false failure feedback.

## 3. Experiment 2: Improvement in Depressive Mood from Self-Positivity Bias Training

### 3.1. Methodology

#### 3.1.1. Participants

Forty college students, 10 men and 30 women, aged 18–25 years (mean age 21 ± 2.3) were randomly selected at Northeast Normal University. We randomly assigned 20 participants to each of the experimental and control groups. One participant in the experimental group failed to answer the questions and was excluded, so there were 39 valid participants. As in experiment 1, the same visual and Raven’s Standard Progressive Matrices criteria were applied.

#### 3.1.2. Experimental Apparatus and Measurement Instruments

In experiment 2, the apparatus and measurement were the same as in experiment 1.

#### 3.1.3. Experimental Procedures

The experiment comprised of three testing phases and three manipulation phases. In the first phase, participants completed a pre-test to measure their baseline level of depressive mood. Next, all participants received false failure feedback. After this, a mid-test was conducted to measure the level of depressive mood induced by the false feedback. Following the mid-test, participants were randomly assigned to either the self-positivity bias training or neutral training condition. After completing the training, a post-test was administered to measure the participants’ level of depressive mood. Lastly, participants were debriefed to remove the negative effects caused by the false feedback, as in experiment 1.

### 3.2. Results

Table 3 gives the means and standard deviations of depressive mood scores between groups and times in Experiment 2. An analysis of variance with repeated measures was conducted on the depressive mood scores for two groups (experimental, control) and three measurement times (pre-test, mid-test, post-test). The result showed a significant main effect for measurement time, *F*(2,74) = 30.58, *p* < 0.001, *η_p_*^2^ = 0.453. The post-hoc analysis found that the scores of depressive mood during the mid-test were significantly higher than those at the pre-test and post-test (*p*s < 0.001), with no statistically significant difference between the pre-test and post-test (*p* = 0.550). The main effect of the group was not significant; *F*(1,34) = 0.48, *p* = 0.493. The interaction effect between measurement time and group was significant, *F*(2,74) = 5.265, *p* < 0.01, *η_p_*^2^ = 0.125. The simple effects analysis revealed that at the pre-test and mid-test, there were no significant differences in depressive mood between the control and experimental groups (*p* = 0.443), and at the post-test, the experimental group showed significantly lower depressive mood scores than the control group (*p* < 0.01). These findings suggested that the self-positivity bias training used in the experimental group was more effective in alleviating depressive mood after failure compared to the control group.

Moreover, in the experimental group, the scores of depressive mood in the mid-test were significantly higher than in the pre-test and post-test (*p* < 0.001), with no significant difference between the post-test and pre-test (*p* = 0.199). These findings suggested that failure feedback can cause an increase in depressive mood, which was alleviated after self-positivity bias training and returned to the initial state. On the other hand, in the control group, the scores of depressive mood in the mid-test were significantly higher on the pre-test (*p* < 0.01) and post-test (*p* = 0.012), and the post-test was significantly higher than the pre-test (*p* = 0.034). These results suggested that the scores of depressive mood significantly increased after failure feedback, and depressive mood was somewhat alleviated after neutral training but did not return to initial levels.

## 4. Discussion

Our primary objective was to investigate the effectiveness of training in improving self-positivity bias. Several previous manipulations have been used to enhance implicit self-positivity bias or implicit self-esteem, but few have been used to enhance explicit self-positivity bias. Based on Maricuțoiu et al. [16], we modified a self-reference task similar to the IAT. Consistent with their findings, the present study found that self-positivity bias training enhanced self-positivity bias and had a greater effect size than the largest one in their three experiments. In the study by Maricuţoiu et al., the maximum effect size was 0.386, and in this study, the maximum effect size was 0.674. Our manipulation was changed from a simple categorization task to a judgment task. This might prompt the participants to recall personal experiences related to their positive traits, prompting them to ask questions such as “Do I possess this positive trait?” and “What memories from my past demonstrate this trait?”. For the purpose of testing the reasons for the enhancement of self-positive bias, we recruited another 25 participants, gave them some self-positive bias training, and asked them the above questions afterward. It was found that 68% of the participants said “yes”, 24% of the participants were “not sure”, and 8% of the participants said “no” for “when judging, I will ask myself ‘do I have this trait?’”; for “When judging, will you recall the facts or events that reflect that you have a certain quality?”, 52% of the participants said “yes”, 28% of the participants said “not sure”, and 20% of the participants said “no”. To some extent, the self-positive bias training task in this study triggered the subjects’ self-reflection and autobiographical memory.

This manipulation is similar to the task used to enhance explicit self-esteem by asking subjects to write about self-positive traits (desirable characteristics possessed or undesirable characteristics lacked) [24]. It is a propositional manipulation and therefore more likely to result in changes in explicit attitudes [25]. In contrast, Maricuţoiu et al.’s study [16] used the Positivity Scale to assess individual positivity about oneself, one’s life, and one’s future. This scale evaluates individuals’ positive self-views rather than positive self-evaluation, but our study utilized a self-reference task as a means of measuring self-positivity bias directly, which was to the greatest extent possible a measure of the effects self-positivity bias training.

The second objective of the present study was to examine the role of enhanced self-positivity bias in alleviating negative emotional distress such as depressive mood. In both experiments, we manipulated negative events through negative feedback to evoke depressive mood in participants, and we trained the participants of the experimental group in self-positivity bias before (experiment 1) and after (experiment 2) the negative events. Both experimental and control groups displayed significantly higher depressive mood scores after the failure event in experiment 1. As a result, the enhanced self-positivity bias in advance in the experimental group did not decrease the depressive mood caused by the failure event, suggesting that the enhanced self-positivity bias was ineffective in combating the depressive mood induced by the failure manipulation. This finding diverges from the results of a comparable study conducted by Dijksterhuis [15], which demonstrated that augmented implicit self-esteem decreased individuals’ susceptibility to failure feedback, leading to diminished negative affective responses following failure. One possible explanation for the discrepancy between our findings and those of Dijksterhuis is that implicit self-esteem and explicit self-positivity bias differ not only at the conscious level but also in their connotations. Additionally, the measurement of emotional reactions to failure may also differ between studies, as Dijksterhuis relied on a single question (“How good do you feel?” on a nine-point scale) to assess mood following negative feedback, while our study used a depressive mood scale to measure the specific current emotional state.

There are two possible explanations for the absence of enhanced self-positivity bias against depressive mood in this study. First, external environmental factors such as life events may have a powerful and pervasive effect on human mood, with success evoking positive emotions and failure inducing negative ones. Mood may be an adaptive response to the evaluation of external stimuli, regardless of an individual’s level of self-perception positivity [2,26]. Second, while the self-positivity bias training increased the level of self-positivity bias in the experimental group, the training effect may have been difficult to detect before negative feedback due to the overall high level of self-positivity bias in normal subjects. Future research should compare the difference in depressive mood between subjects with high and low self-positivity bias after experiencing negative events to reveal the potential impact of high self-positivity bias on lowering depressive mood.

In experiment 2, we found that the depressive mood in the self-positivity bias training group was significantly lower than prior to training, as well as lower than it was prior to failure manipulation, compared with the neutral training group. Thus, self-positivity bias training appears to have a positive effect on depression induced by failure. In contrast, Maricuțoiu et al. found that enhanced explicit self-esteem and explicit positivity had no effect on psychological well-being (as measured via a short version of the Anxiety-Depression-Stress Scale, DASS). Maricuțoiu et al. did not measure the participants’ mental health before the training task [16]. As a result, the additional variables that were not controlled may have been mixed with the experimental variables. This could explain why the study found differences in mental health between the experimental and control groups only after the participants in the experimental group completed a modified self-reference task and the participants in the control group completed a categorization judgment task for positive and negative stimuli.

Furthermore, experiment 2 revealed a positive impact of enhanced self-positivity bias on depressive mood induced by failure. The underlying reason for this finding may stem from self-affirmation theory [7], which posits that threatening information can undermine individuals’ positive self-evaluation. Consequently, individuals may protect their self-concept by affirming their self-worth and traits in domains unrelated to the threatening information, which helps to counteract the threat and maintain their sense of self-worth. In the present study, the failure event served as threatening information that could damage individuals’ positive self. However, self-positivity bias training offered participants a platform to recognize and affirm themselves, which facilitated the restoration of their positive self-image through self-positivity training. As a result, this training helped to mitigate the depressive mood caused by failure.

The feedback after the Raven reasoning test was “You have failed this test and your correct score is below the average for university students (the average Chinese university student gets seven questions correct)”. The failure of intelligence tests can result in negative emotions such as anxiety and depression [23]. Although we did not find direct evidence that failure events may negatively affect self-evaluation, some studies have shown that the academic self-worth of subjects with high self-esteem is reduced when they are required to recall recent failed academic events [27]. To some extent, this indicates that failure events related to ability will negatively affect an individual’s perception of their worth. Therefore, we believe that the experience of failure can also be viewed as a threat to an individual’s evaluation.

Through the manipulation of false failure feedback, we simulated the process of depressive emotions in healthy individuals who experienced negative life events in their daily lives. Depression is a temporary emotional state reflected in a depressive mood, which may be alleviated by spending a certain amount of time experiencing it without external intervention. A temporary depression will accumulate and precipitate, becoming a relatively long-lasting and stable depression when the individual experiences multiple negative events in a short period of time or if the individual has a high level of neuroticism and a low level of social support available. Numerous studies have shown that life events can predict levels of depression [28,29]. Furthermore, factors such as self-efficacy [30], hope [31], resilience [32], and others have been found to contribute to the prevention of depression in the context of stressful life events. These findings suggest that in the future, it may be possible to enhance these protective factors to prevent depression triggered by stressful life events.

In addition, attentional bias modification training (ABMT) can improve a depressed individual’s mood if an individual already exhibits depressive symptoms or suffers from a depressive disorder [33]. ABMT is a computer-based cognitive-behavioral therapy (CBT) that is based on Beck’s cognitive theory. In Beck’s theory, individuals with depression possess a negative self-schema, which when activated leads to negative cognitions, which exacerbate depressive symptoms [34]. Negative cognition is primarily associated with attention, memory, and interpretational biases toward negative information in depressed individuals. Through its programmatic training, ABMT aims to reduce depressed individuals’ attention bias toward negative information. Therefore, ABMT primarily focuses on the automatic processing of information, such as increasing attention to positive information and decreasing attention to negative information [35]. There is still a need for further validation to determine whether ABMT alters negative self-schema as well. Compared with ABMT, self-positive bias training implemented in this study is more likely to alter negative self-schema in individuals. Several researchers argue that self-positive bias is a crucial component of Beck’s cognitive theory of depression [36]. It is also intended to enhance individuals’ self-perception and self-evaluation through self-positive bias training.

Additionally, a number of studies have demonstrated that self-positivity bias is closely associated with depression [37,38]. For example, participants with high levels of depressive symptoms have a lower self-positivity bias [36] and are more likely to endorse negative words [39], and individuals with depressive characteristics do not exhibit implicit self-positivity bias in their cognitive processing [12]. As depression scores increased, healthy college students deemed negative words to be self-relevant in a self-evaluation context, indicating that depression affected negative self-evaluations and self-positivity biases [40]. Another study found that depression scores were negatively correlated with positive self-evaluation, positively correlated with negative self-evaluation, and negatively correlated with depression severity in adolescents [41]. Given all the findings demonstrating that enhanced self-positivity bias can improve depressive mood, future research might enable self-positivity bias training to be applied to individuals with non-clinical depression symptoms as well as clinically depressed individuals.

## 5. Conclusions

The findings demonstrate that failure events can lead to depression among healthy college students, while self-positivity bias training can enhance self-positivity bias through positive self-reflection and autobiographical memory stimulation. Even though self-positive bias did not directly counteract the depressive mood triggered by failures, it demonstrated a significant positive impact on depression mood. The causal relationship between self-positive bias and depression has been established, partially supporting Beck’s cognitive theory. These results suggest that self-positive bias training holds potential for future use in addressing non-clinical depressive symptoms and as an adjunctive treatment for clinical depression.

## Figures and Tables

**Table 1:** Pre-test and post-test scores of self-positivity bias between groups (*M ± SD*).

		Positive Words Like Me	Negative Words Like Me	Self-Positivity Bias
Experimental Group	Pre-test	179 ± 26	115 ± 26	64
	Post-test	185 ± 21	102 ± 25	83
Control Group	Pre-test	182 ± 18	105 ± 25	77
	Post-test	179 ± 18	104 ± 28	74

**Table 2:** Depressive mood between groups and times in experiment 1 (*M ± SD*).

	Pre-Test	Mid-Test	Post-Test
Experimental Group	6.6 ± 3.56	6.9 ± 3.49	11.9 ± 6.83
Control Group	8.5 ± 4.36	8.5 ± 4.82	12.9 ± 6.87

**Table 3:** Depressive mood between groups and times in experiment 2 (*M ± SD*).

	Pre-Test	Mid-Test	Post-Test
Experimental Group	7.6 ± 3.34	14.2 ± 5.36	6.2 ± 3.12
Control Group	7.8 ± 6.19	12.8 ± 5.96	10.0 ± 4.90

## Data Availability

The data presented in this study are available on request from the corresponding author.
